# Wellens’ syndrome following severe COVID-19 infection, an innocent coincidence or a deadly association: two case reports

**DOI:** 10.1186/s12872-023-03137-7

**Published:** 2023-02-24

**Authors:** Georges Khattar, Jennifer Hallit, Carolla El Chamieh, Elie Bou Sanayeh

**Affiliations:** 1 Faculty of Medicine and Medical Sciences, Holy Spirit University of Kaslik (USEK), Jounieh, Lebanon; 2 Notre Dame des Secours University Hospital, Jbeil, Lebanon; 3 Department of Pediatrics, American University of Beirut Medical Center, Beirut, Lebanon; 4 Department of Internal Medicine, American University of Beirut Medical Center, Beirut, Lebanon

**Keywords:** COVID-19, Wellens’ syndrome, Acute coronary syndrome, Atherosclerosis, COVID-19 complications

## Abstract

**Background:**

Coronavirus disease 2019 (COVID-19) has been associated with late-onset cardiovascular complications primarily due to a hypercoagulable state. Its association with Wellens’ syndrome, which reflects a stenosis in the proximal left anterior descending coronary artery, is not well established. We present two cases diagnosed with this syndrome following their COVID-19 acute phase despite taking adequate anticoagulation.

**Case presentation:**

We present two patients with incidental electrocardiography (ECG) showing the typical Wellens’-related changes, with an underlying severe triple-vessel coronary artery disease a few weeks following a severe COVID-19 infection associated with high inflammatory markers. The stenotic lesions were diagnosed by cardiac catheterization, and both patients underwent Coronary Artery Bypass Grafting successfully. Notably, patients’ baseline ECGs were normal, and they were maintained on Rivaroxaban 10 mg following their viral illness.

**Conclusion:**

Despite advances in the preventive measures for COVID-19 complications, its pathophysiologic impact on vasculature and atherosclerosis is still incompletely understood. Further clinical trials must be conducted to study this association between Wellens’ syndrome and this virus to prevent life-threatening complications.

## Background

Wellens’ syndrome, also known as “Left Anterior Descending (LAD) coronary artery T-wave syndrome” or “acute coronary T-wave syndrome,” represents a pre-infarction state due to a severe narrowing of the LAD associated with a history of chest pain. However, patients are typically asymptomatic at the time of diagnosis. Therefore, it is a relatively underdiagnosed entity characterized by distinctive electrocardiographic changes that should raise the suspicion of acute coronary syndrome (ACS) in the appropriate clinical settings [1]. Early recognition of these changes and urgent intervention are vital to avoid the progression into a significantly irreversible myocardial loss.

With the continuous surge of Coronavirus disease 2019 infections (COVID-19), it is vital always to consider this unfamiliar syndrome, given the significant association between this virus and late thrombotic cardiovascular events such as myocardial infarctions (MI), strokes, limb ischemia, etc. [2] Therefore, a suitable patient history should always include detailed COVID-19 infection and vaccination status information.

The ongoing interest in the novel COVID-19 has revealed many associations. However, to our knowledge, there are only two reported cases in the literature describing Wellens’ syndrome during a coronavirus infection [3, 4] (1,2)(1,2). We present two cases in which incidental electrocardiography (ECG) showed typical Wellens-related changes, with an underlying severe triple-vessel coronary artery disease (CAD) following a severe COVID-19 infection.

## Case presentation

**Fig. 1:**
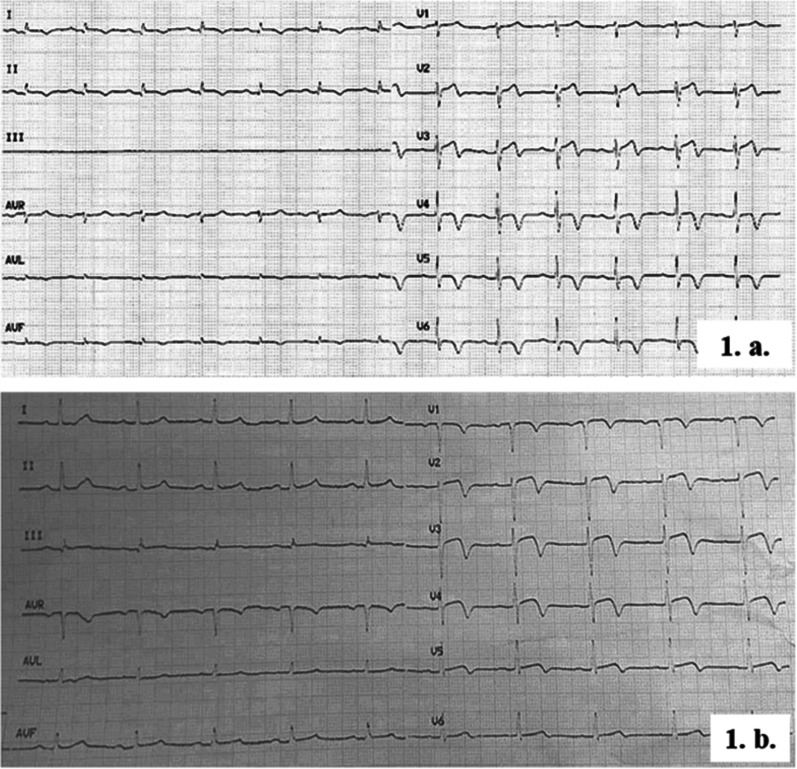
**1.a.** First patient’s ECG: Sinus rhythm with a 1 mm ST-segment elevation and a biphasic T-wave in V2 and V3 precordial leads; T-wave inversion in V4, V5, and V6 leads. Absence of pathological Q-wave in these leads preserved R-wave progression from V1 to V6. **1.b.** Second patient’s ECG: Sinus rhythm with a 2 mm ST-segment elevation and a biphasic T-wave on V2, V3, V4, and V5 precordial leads. Absence of pathological Q-wave in these leads and preserved R-wave progression from V1 to V6

The first patient is a 55-year-old Lebanese man admitted for right carpal tunnel release. A pre-operative routine ECG showed sinus rhythm with a 1 mm ST-segment elevation, biphasic T-wave in V2-V3 precordial leads, and T-wave inversion in V4-V5-V6 leads as well as leads II and aVF (Fig. 1.a). The second patient is a 62-year-old Lebanese female admitted for a urinary tract infection. Her baseline ECG showed sinus rhythm with a 2 mm ST-segment elevation and a biphasic T-wave in V2-V3-V4-V5 precordial leads (Fig. 1.b). Both patients reported a history of chest pain on exertion during the weeks prior to their current presentation. No cardiovascular risk factors or family history of CAD were noted except for heavy smoking in both patients. Both patients were vaccinated against COVID-19, more than six months prior to the current presentation, with two doses of Pfizer-BioNTech and 1 dose of Johnson & Johnson's Janssen, respectively.

They both suffered from a severe COVID-19 infection requiring oxygen supplementation via a non-rebreather facemask. They had high inflammatory markers: C-Reactive Protein (CRP) > 150 mg/L, Ferritin > 1000 mcg/L, and D-dimers reaching 3560 ng/mL and 4200 ng/mL, respectively. They had normal baseline ECG and troponin levels. An adequate evaluation revealed no pulmonary embolism or deep venous thrombosis. High doses of intravenous corticosteroids were administered for ten days. After cessation of oxygen supplementation and a total hospital stay of 19 and 14 days, respectively, both patients were discharged on Rivaroxaban 10 mg for 6 weeks.

**Fig. 2:**
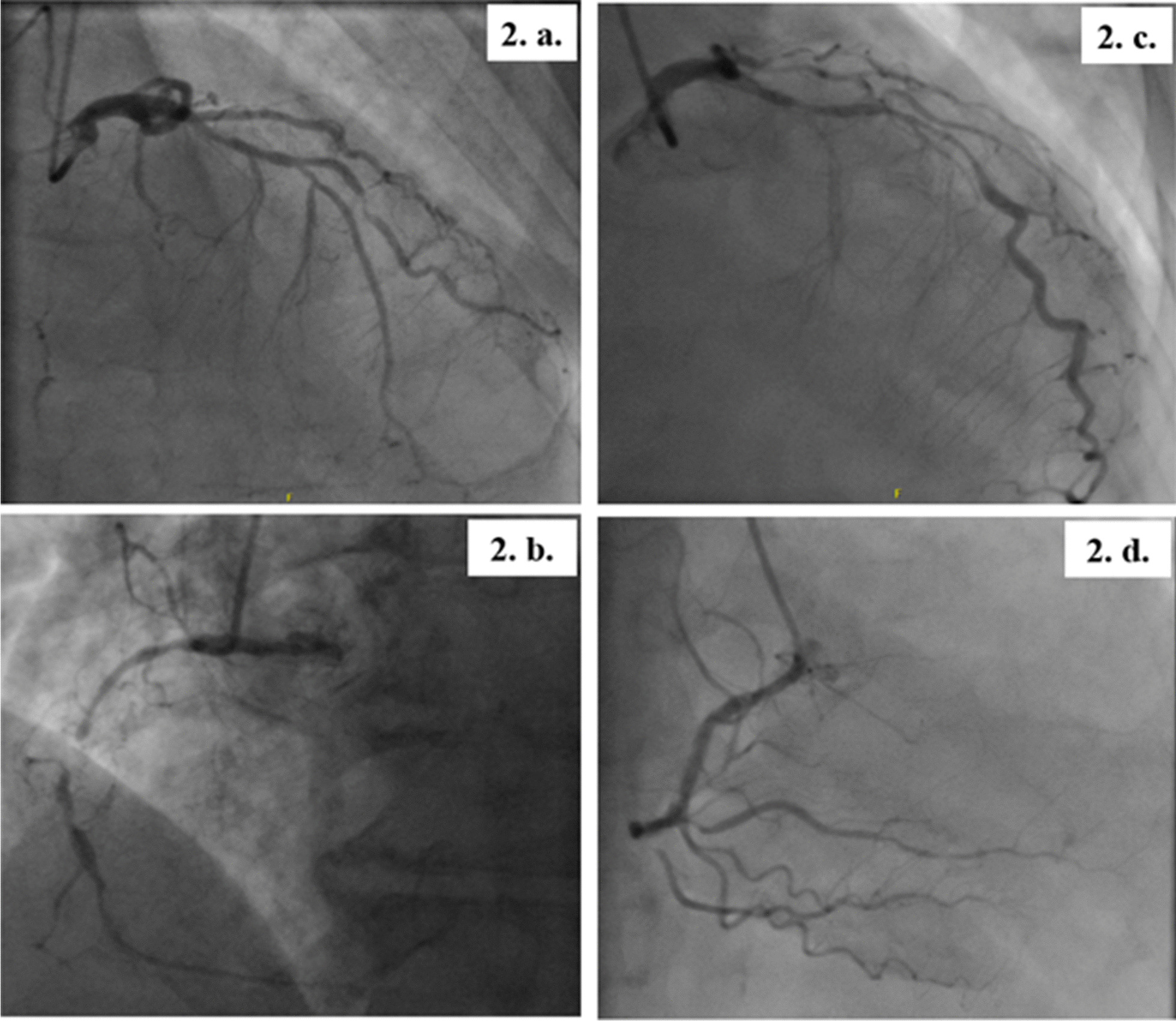
The patients’ coronary angiography- part 1. *Patient 1* **2. a.** Long mid-segment of 70–80% stenosis in the LAD, 50% ostial stenosis in the first diagonal coronary artery (DCA), mid-segment 95% thrombotic stenosis in the Second DCA, and proximal 70–80% stenosis in the circumflex coronary artery (Cx). **2. b.** Mid-segment chronic total occlusion of the right coronary artery (RCA) with bridging collaterals. The posterior descending artery (PDA) and posterior left ventricular artery (PLV) fill retrogradely from collaterals arising from the LAD septal branches. *Patient 2* **2. c.** Ostial-mid long segment of 80–90% stenosis in the LAD, sub-total occlusion of the First DCA and Cx, and proximal 80% stenosis in the first obtuse marginal (OM1). **2. d.** 80% stenosis of the mid RCA and ostial 80% stenosis of the PDA. The PLV has a chronic total occlusion proximally and is receiving collaterals distally from the distal PDA

**Fig. 3:**
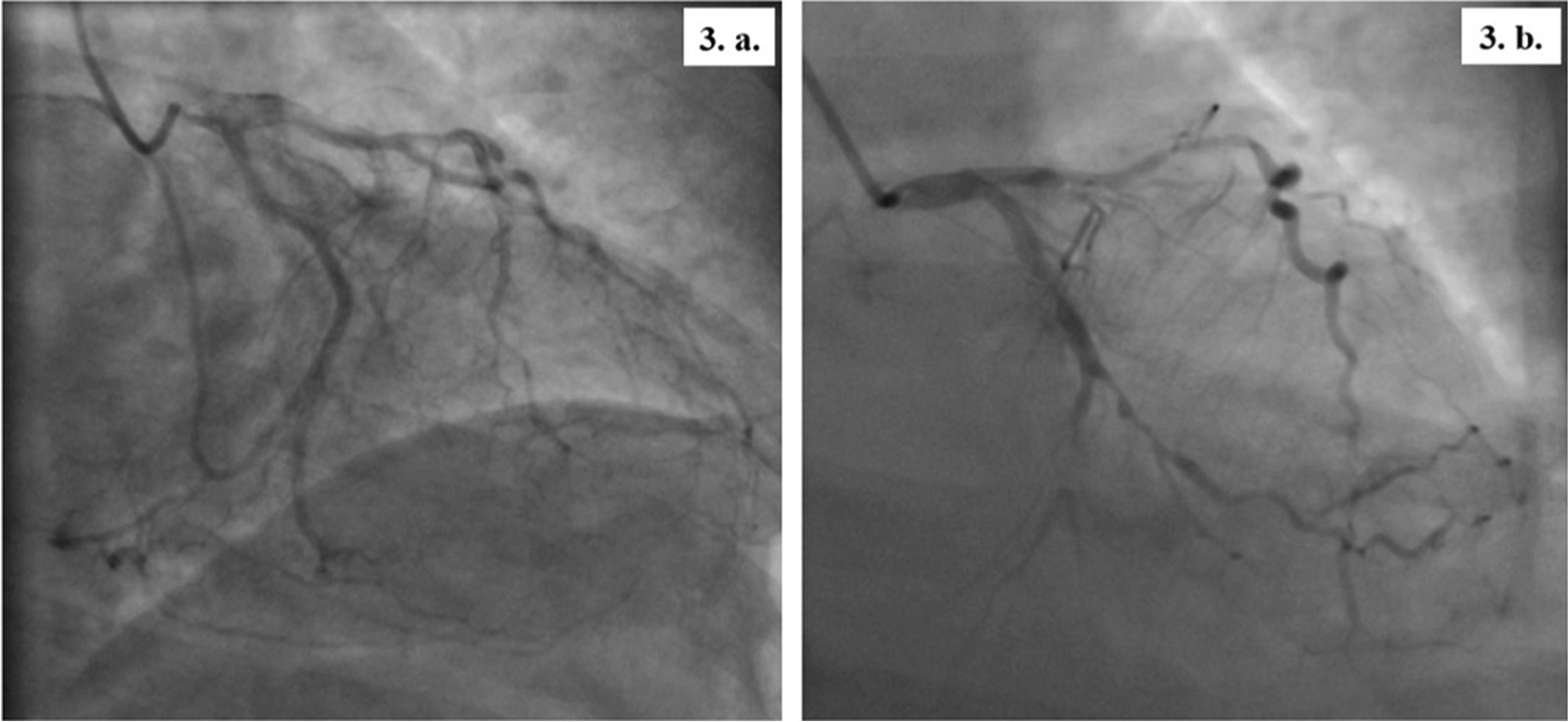
The patients’ coronary angiography- part 2. *Patient 1*:**3. a.** The Circumflex coronary artery has a proximal 70–80% stenosis with diffuse atherosclerosis. *Patient 2* **3. b.** The circumflex coronary artery has mild diffuse disease with subtotal occlusion in the small mid-distal circumflex proper just after the bifurcation of Obtuse Marginal 1 (OM1). OM1 is medium size vessel with a proximal 80% stenosis followed by diffuse disease

The patients presented at 8 and 9 weeks, respectively, after discharge. They both were hemodynamically stable during the current evaluation and pain-free with an unremarkable systemic examination. Cardiac biomarkers (creatine kinase myocardial band-CPK-MB and troponin T) and basic blood parameters were normal. In both patients, further cardiac evaluation with transthoracic echocardiography revealed wall motion abnormalities in the LAD territory with a left ventricular ejection fraction (LVEF) of 45–49% in the first patient and 35–39% in the second one. At last, we found in both of them on coronary angiograms a severe calcified triple-vessel disease (Fig. 2) that is not amenable to percutaneous coronary intervention (PCI) (Fig. 2a and c). Patients were started on Aspirin, unfractionated Heparin and adequate heart failure pharmacotherapy. They were then referred for surgical revascularization via coronary artery bypass grafting (CABG). The operations were successfully performed on the third and fifth day of admission, respectively. Repeated ECGs one month following the surgery showed complete resolution of the abovementioned pathological changes (Fig. 3).

## Discussion

Wellens’ syndrome is an independent risk factor for serious cardiovascular events and is typically associated with coronary artery syndrome [1]. The two presented cases suggest a possible association between this syndrome and COVID-19 infection.

Wellens’ syndrome is usually associated with critical stenosis in the proximal LAD when first described; however, due to the confusion in segmentation between societies, the same presentation has been described with mid-LAD stenosis [5]. This syndrome consists of a post-reperfusion state with a 75% probability of re-occlusion and extensive anterior wall myocardial infarction within one week of diagnosis [6, 7]. Therefore, early diagnosis and prompt management can be life-saving. The first step in its diagnosis is the recognition of the unique ECG changes (Wellen’s changes) that are thought to be secondary to myocardial edema resulting from repetitive episodes of transmural ischemia and reperfusion [1]. The characteristic changes occur in two forms: type A with biphasic T-wave changes (with initial positivity and terminal negativity), mainly in V2 and V3 precordial leads, and type B with deep T-wave inversion affecting the same leads (but may also be seen in other precordial leads) [1]. In both types, pathological Q-waves in precordial leads should not be present, minimal ST-segment changes might be seen, and R-wave progression should be preserved. Both types A and B might be seen in the same ECG, as in our first patient [6]. Clinically, it is required to have a recent history of angina with a resolution of pain at evaluation time [8]. Thus, Wellen’s syndrome can only be diagnosed when having the distinctive Wellen’s ECG changes associated with the typical clinical picture [8]. Furthermore, Wellen’s ECG pattern was also spotted in inferior leads in Patient 1, which might also reflect the severe mid-RCA stenosis; an occurrence described previously by Chioncel et.al [9]. Biologically, cardiac biomarkers can be falsely reassuring, with only 12% of patients having elevated cardiac enzymes [8]. Once the diagnosis is suspected, it is recommended to avoid stress tests due to the high risk of myocardial injury progression and sudden cardiac death. Thus, the best next step would be coronary angiography to confirm the diagnosis and exclude other non-CAD causes of Wellens’ or Pseudo-Wellens’ (false positive ECG appearance of Wellens syndrome), such as vasospasm (secondary to COVID-19, cocaine, marijuana, or phencyclidine), coronary artery fistula, coronary vasculitis, sepsis, pulmonary embolism, or even acute cholecystitis [1, 10–15]. At last, if Wellens’ syndrome diagnosis is confirmed, optimal treatment would be via urgent PCI or CABG to restore adequate blood flow; otherwise, it would be treating the underlying cause.

Aside from causing hypoxic injury to multiple organs leading to a type 2 MI, COVID-19 has also been described as a prothrombotic infection that might induce acute limb ischemia, stroke, myocardial infarction, deep venous thrombosis, and pulmonary embolism. This could be attributed to the impaired activation of the Angiotensin-Converting Enzyme type 2 (ACE-2) receptors, with subsequent endothelial injury [2, 15, 16]. However, most of those complications have been described mainly during the acute infectious or sometimes convalescent phases [17]. This was due to the direct inflammatory changes to the vascular wall following the cytokine storm, which also, if severe, can cause plaque rupture [15]. Adequate prophylactic anticoagulation, similar to what was given to our patients, could help prevent such life-threatening events [17].

Nevertheless, aside from the prothrombotic state, Wellens’ syndrome and ACS share the exact pathophysiology with increased atherosclerotic plaque size and subsequent plaque instability and rupture [1]. An association between COVID-19 and plaque instability has been suggested, with the possible mediating impact of the inflammatory storm [4, 16]. Eventually, the inflammation caused by the release of Interleukins 1–6-32–34, TNF alpha, etc., and activation of multiple intracellular pathways (through CD-147 and NLRP3 inflammasome, etc.), may lead to rapid unstable plaque formation even after resolution of the infection [16, 18, 19]. Moreover, despite decreasing inflammation, high doses of glucocorticoids during the severe COVID-19 disease could also aggravate plaque instability [20]. Multiple other theories and mechanisms have been described as contributors to the fastening of atherogenesis. Subsequently, despite the controversial evidence, Statins’ pleiotropic effect might have a promising effect in plaque stabilization in selected patients with severe inflammation [15, 16].

Based on the two presented cases, it is unclear whether we should consider a previous COVID-19 infection as a risk factor for Wellens’ syndrome, given that both patients were heavy smokers, predisposing them to cardiovascular diseases. Subsequently, further in vitro studies and randomized trials are required to establish a more explicit association between COVID-19 and atherosclerosis progression.

Meanwhile, physicians should remain vigilant while treating post-COVID-19 patients. Given the large disease incidence, it would be impossible to conduct an ECG on all newly infected patients. However, close follow-up, and adequate counseling and education regarding ACS symptoms must be given for patients with multiple cardiac risk factors and comorbidities as COVID-19 might contribute to the rapid progression of atherosclerosis and increase in the risk of plaque instability.

## Data Availability

Data sharing is not applicable to this article as no datasets were generated or analyzed during the current study.

## Abbreviations

LAD: Left anterior descending
ACS: Acute coronary syndrome
COVID-19: Coronavirus disease 2019
MI: Myocardial infarction
ECG: Electrocardiography
CAD: Coronary artery disease
CRP: C-reactive protein
CK-MB: Creatine kinase myocardial band
LVEF: Left ventricular ejection fraction
PCI: Percutaneous coronary intervention
CABG: Coronary artery bypass grafting
ACE-2: Angiotensin-converting enzyme type 2
